# The *ESR1* (6q25) Locus Is Associated with Calcaneal Ultrasound Parameters and Radial Volumetric Bone Mineral Density in European Men

**DOI:** 10.1371/journal.pone.0022037

**Published:** 2011-07-07

**Authors:** Kate L. Holliday, Stephen R. Pye, Wendy Thomson, Steven Boonen, Herman Borghs, Dirk Vanderschueren, Evelien Gielen, Ilpo T. Huhtaniemi, Judith E. Adams, Kate A. Ward, Gyorgy Bartfai, Felipe Casanueva, Joseph D. Finn, Gianni Forti, Aleksander Giwercman, Thang S. Han, Krzysztof Kula, Fernand Labrie, Michael E. J. Lean, Neil Pendleton, Margus Punab, Frederick C. W. Wu, Terence W. O'Neill, and the EMAS study group

**Affiliations:** 1 Arthritis Research UK Epidemiology Unit, The University of Manchester, Manchester Academic Health Science Centre, Manchester, United Kingdom; 2 Leuven University Division of Geriatric Medicine, Katholieke Universiteit Leuven, Leuven, Belgium; 3 Leuven University Center for Metabolic Bone Diseases, Katholieke Universiteit Leuven, Leuven, Belgium; 4 Department of Andrology and Endocrinology, Katholieke Universiteit Leuven, Leuven, Belgium; 5 Department of Surgery and Cancer, Imperial College London, Hammersmith Campus, London, United Kingdom; 6 Clinical Radiology, Imaging Science and Biomedical Engineering, The University of Manchester, and The Royal Infirmary, Manchester Academic Health Science Centre, Manchester, United Kingdom; 7 MRC Human Nutrition Research, Elsie Widdowson Laboratory, Cambridge, United Kingdom; 8 Department of Obstetrics, Gynaecology and Andrology, Albert Szent-Gyorgy Medical University, Szeged, Hungary; 9 Department of Medicine, Santiago de Compostela University, Complejo Hospitalario Universitario de Santiago (CHUS), CIBER de Fisiopatología Obesidad y Nutricion (CB06/03), Instituto Salud Carlos III, Santiago de Compostela, Spain; 10 Andrology Research Unit, Developmental and Regenerative Biomedicine Research Group, The University of Manchester, Manchester Academic Health Science Centre, Manchester Royal Infirmary, Manchester, United Kingdom; 11 Endocrinology Unit, Department of Clinical Physiopathology, University of Florence, Florence, Italy; 12 Reproductive Medicine Centre, Skåne University Hospital, University of Lund, Malmo, Sweden; 13 Department of Endocrinology, University College London, London, United Kingdom; 14 Department of Andrology and Reproductive Endocrinology, Medical University of Lodz, Lodz, Poland; 15 Laboratory of Molecular Endocrinology and Oncology, Laval University, Quebec City, Quebec, Canada; 16 Department of Human Nutrition, University of Glasgow, Glasgow, Scotland; 17 School of Community Based Medicine, The University of Manchester, Salford Royal NHS Trust, Salford, United Kingdom; 18 Andrology Unit, United Laboratories of Tartu University Clinics, Tartu, Estonia; University of Hong Kong, Hong Kong

## Abstract

**Purpose:**

Genome-wide association studies (GWAS) have identified 6q25, which incorporates the *oestrogen receptor α* gene (*ESR1*), as a quantitative trait locus for areal bone mineral density (BMD_a_) of the hip and lumbar spine. The aim of this study was to determine the influence of this locus on other bone health outcomes; calcaneal ultrasound (QUS) parameters, radial peripheral quantitative computed tomography (pQCT) parameters and markers of bone turnover in a population sample of European men.

**Methods:**

Eight single nucleotide polymorphisms (SNP) in the 6q25 locus were genotyped in men aged 40–79 years from 7 European countries, participating in the European Male Ageing Study (EMAS). The associations between SNPs and measured bone parameters were tested under an additive genetic model adjusting for centre using linear regression.

**Results:**

2468 men, mean (SD) aged 59.9 (11.1) years had QUS measurements performed and bone turnover marker levels measured. A subset of 628 men had DXA and pQCT measurements. Multiple independent SNPs showed significant associations with BMD using all three measurement techniques. Most notably, rs1999805 was associated with a 0.10 SD (95%CI 0.05, 0.16; p = 0.0001) lower estimated BMD at the calcaneus, a 0.14 SD (95%CI 0.05, 0.24; p = 0.004) lower total hip BMD_a_, a 0.12 SD (95%CI 0.02, 0.23; p = 0.026) lower lumbar spine BMD_a_ and a 0.18 SD (95%CI 0.06, 0.29; p = 0.003) lower trabecular BMD at the distal radius for each copy of the minor allele. There was no association with serum levels of bone turnover markers and a single SNP which was associated with cortical density was also associated with cortical BMC and thickness.

**Conclusions:**

Our data replicate previous associations found between SNPs in the 6q25 locus and BMD_a_ at the hip and extend these data to include associations with calcaneal ultrasound parameters and radial volumetric BMD.

## Introduction

Oestrogens have positive effects on the development and maintenance of the skeleton. These effects include the regulation of bone turnover, acquisition of peak bone mass and inhibition of bone loss. The majority of previous work has focussed on oestrogens and the female skeleton, but more recent evidence suggests that oestrogens are also required for the maintenance of bone health in men [1]–[9]. The effects are mediated through binding to specific oestrogen receptors (ER), which belong to the nuclear hormone receptor superfamily and are expressed in a number of cell types including osteoblasts, osteoclasts and bone marrow stromal cells [10], [11]. Two functional oestrogen receptors have been identified so far, ERα and ERβ. ERα appears to be the major receptor, having a prominent effect on bone metabolism [12].

The human gene for ERα, *ESR1*, is located on chromosome 6q25. Three polymorphisms in *ESR1* have been widely studied to date; two restriction fragment length polymorphisms in intron 1, rs9340799 (*Xba*I) and rs2234693 (*Pvu*II), and the (TA)_n_ variable number tandem repeat in the promoter region of the gene. Several previous studies have found associations between these polymorphisms and osteoporosis related phenotypes, but the results are somewhat conflicting possibly due to small sample sizes, variation in study design and differences between the populations studied [12]. In a large-scale meta-analysis of over 18,000 subjects, an association was observed between rs9340799 and fracture risk in women that was independent of areal bone mineral density (BMD_a_), however, there was no association between rs2234693 or the TA repeat and fracture and none of the polymorphisms were associated with BMD_a_ [13].

In recent years, genome-wide association studies (GWAS) have been utilised to try to elucidate genetic predictors of bone health using a hypothesis-free approach. One of the first GWAS, conducted by Styrkarsdottir et al [14], reported a strong association between the 6q25 region and dual energy x-ray absorptiometry [DXA] hip and lumbar spine (LS) BMD_a_, which has subsequently been replicated in independent populations [15]. This locus includes the 5′ end of *ESR1* and a gene of unknown function (*C6orf97*) and is independent (r^2^<0.1, HapMap CEPH) of the aforementioned widely studied polymorphisms. In a recent meta-analysis, which utilised data from five GWAS and included over 19,000 subjects, the locus at 6q25 was one of the region's most strongly associated with LS and femoral neck (FN) BMD_a_ [16].

The majority of genetic association studies of bone health have focused on post-menopausal women with many of the large cohorts consisting of predominantly females, therefore the role of the 6q25 locus in male populations is less well characterised. Previous work has understandably focused on areal bone density (measured by DXA) at the two most clinically relevant osteoporotic sites (hip and spine). However, there are limitations in the assessment of bone health using DXA, one of the most important being that BMD_a_ is influenced by structural and geometric parameters such as perisosteal expansion, cortical density, cortical thickness, and trabecular number and thickness [17]. For example, it is thought that periosteal expansion reflects skeletal growth whereas cortical density reflects bone remodeling [18]. More recently, techniques such as peripheral quantitative computed tomography (pQCT) have been developed to allow assessment of the structural and geometric components of bone. However the influence of the 6q25 locus on pQCT parameters is not clear. Futhermore, the influence the locus may have on bone remodeling as measured by serum markers of bone formation and resorption has yet to be determined. These parameters may have distinct genetic determinants, knowledge of which could further our understanding of the biological mechanisms underlying bone health. In addition, DXA is not always practical, particularly in large scale multi-site research studies. Thus techniques such as quantitative ultrasound (QUS), which prospective studies have shown to predict osteoporotic fracture as well as DXA [19]–[22], have been utilised to assess bone health. The influence the 6q25 locus has on QUS parameters is not known.

The European Male Ageing Study (EMAS) is a large multicentre population based study of ageing in middle aged and elderly European men which collected an extensive range of clinical, biochemical and other health information. We used data from EMAS to determine if the associations previously observed between SNPs in 6q25 and DXA BMD_a_ at the hip and spine could be replicated. We then examined if 6q25 was associated with bone health as measured by QUS at the calcaneus, pQCT at the radius and serum markers of bone turnover.

## Methods

### Ethics statement

Ethical approval for the study was obtained in accordance with local institutional requirements in each centre: Florence (Ethical Committee of the Azienda Ospedaliera Careggi & University of Florence), Leuven (Commissie Medische Ethiek UZ Gasthuisberg & KU Leuven), Lodz (Bioethical Committee of Medical University of Lodz for Human Studies), Malmö (Ethical Committee of Lund University), Manchester (North West Multi Centre Ethical Research Committee, University of Manchester & CMMCUH), Santiago de Compostela (Comité Ético de Investigación Clínica de Galicia & Universidad de Santiago de Compostela), Szeged (Human Investigation Review Board, University of Szeged) and Tartu (Ethical Committee, Medical University of Tartu). All subjects provided written informed consent. Approval for the genetic analysis described here was obtained for seven of the eight centres. Therefore, analysis was restricted to subjects from these seven centres (all centres except for Malmö, Sweden).

### Subjects

The subjects included in this analysis were recruited for participation in EMAS. Detailed methods have been described previously [23]. Briefly, men were recruited from population based sampling frames in 8 European centres: Florence (Italy), Leuven (Belgium), Lodz (Poland), Malmö (Sweden), Manchester (UK), Santiago de Compostela (Spain), Szeged (Hungary), Tartu (Estonia). Stratified random sampling was used with the aim of recruiting equal numbers of men in each of four 10-year age bands: 40–49 years, 50–59 years, 60–69 years, and 70 years and over.

### Blood sampling and DNA extraction

Fasting blood samples were collected before 10 a.m. and maintained at 0 to 4°C during all stages of the separation procedures. After allowing whole blood samples to clot for 1 hour, they were centrifuged at 3000 rev/min. The aliquots of plasma and buffy coat were stored at −80°C. DNA was extracted from leukocytes using standard phenol-chloroform extraction and stored at −80°C prior to further analysis. Serum was also separated immediately after phlebotomy and stored at −80°C. Each serum sample was assayed for oestradiol (E_2_) and sex-hormone binding globulin (SHBG). SHBG was measured by Modular E170 platform electrochemiluminescence immunoassay (Roche Diagnostics, Mannheim, Germany) at a single laboratory (General Laboratory, Azienda Ospedaliero-Universitaria Careggi, Florence, Italy) as described previously [24]. E_2_ was measured using gas chromatography-mass spectroscopy (GC-MS) [25]. Free (non-SHBG-bound) E_2_ levels were derived using the total hormone measurement and levels of SHBG and albumin using mass action equations and association constants [26] [27].

### SNP selection and genotyping

SNPs in the *ESR1* locus (6q25) associated with LS and/or Hip BMD_a_ in the GWAS conducted by Styrkarsdottir et al (2008) were selected for genotyping (for results in Styrkarsdottir et al (2008) see Table S1). The selected SNPs and proxies (r^2^ = 1) were genotyped using Sequenom MassARRAY technology in accordance with the manufacturer's instructions. Sample and assay quality control thresholds were set to 90%. SNPs were tested for deviation from Hardy-Weinberg Equilibrium (HWE) and excluded from the analysis if p<0.05 in the total population. Linkage disequilibrium was examined using Haploview [28].

### Bone assessments

#### Quantitative ultrasound (QUS)

Quantitative ultrasound (QUS) of the calcaneus was performed using the Sahara Clinical Sonometer (Hologic Inc, Bedford, MA, USA) using a standardized protocol. Each centre used the same machine model, and each calibrated daily with the physical phantom provided by the manufacturer. Outputs included broadband ultrasound attenuation (BUA), speed of sound (SOS) and a machine derived parameter: calcaneal bone mineral density (eBMD) in g/cm^2^, eBMD = 0.002592×(BUA+SOS)−3.687. To establish the short-term precision of the method in this population, duplicate measurements were performed in 20 randomly selected cohort members in one of the centres (Leuven, Belgium). The in vivo CVs were 2.8%, 0.3%, 3.4% for BUA, SOS and eBMD respectively.

#### Dual-energy x-ray absorptiometry (DXA)

Areal bone mineral density (BMD_a_) scans were carried out in two centres (Manchester and Leuven). Both sites used dual-energy x-ray absorptiometry (DXA) QDR 4500A devices from the same manufacturer (Hologic Inc, Bedford, MA, USA). BMD_a_ was measured at the lumbar spine (L1 to L4) and proximal femur (total region). All scans and analysis were performed by trained and certified DXA technicians. The CVs for LS and total hip BMD_a_ were 0.57% and 0.56% in Leuven, respectively, and the CVs for both LS and total hip were 0.97% in Manchester.

#### Peripheral Quantitative Computed Tomography (pQCT)

Pexripheral QCT was performed in the same two centres as the DXA measurements. The non-dominant radius was measured using an XCT-2000 scanner (Stratec, Pforzheim, Germany) in each centre following the manufacturer's standard quality assurance procedures. Total and trabecular volumetric BMD (vBMD) (mg/mm^3^), and bone cross-sectional area (mm^2^) were measured at the distal radius (4%) (voxel size 0.4 mm). Cortical vBMD (mg/mm^3^); total, cortical and medullary area (mm^2^); cortical thickness (mm); and stress strain index (SSI) (mm^3^) were measured at the midshaft radius (50%) (voxel size 0.6 mm).

The European Forearm Phantom (EFP) was measured for cross-calibration between the two centres; 10 repeat measurements were taken in slices 1–4. The differences were less than precision error for total, trabecular and cortical vBMD, and cortical area; therefore, no cross-calibration was performed between the two centres. The short term precision of 2 repeat measurements with repositioning were: total vBMD 2.1% and 1.3%; trabecular vBMD 1.27% and 1.42%; cortical vBMD 0.77% and 0.71%; and cortical area 2.4% and 1.3%; in Manchester (n = 22) and Leuven (n = 40), respectively.

#### Bone turnover markers

To assess bone resorption, serum beta C-terminal telopeptide cross-linked telopeptide (β-cTX) was measured on the Elecsys 2010 automated analyzer (Roche Diagnostics GmbH, Mannheim, Germany) using the ß-Crosslaps/serum reagents [29]. This assay is specific for cross-linked ß-isomerized type I collagen C-telopeptide fragments and uses two monoclonal antibodies, each recognizing the Glu-Lys-Ala-His-ßAsp-Gly-Gly-Arg peptide (Crosslaps antigen). The intra-assay CV evaluated by repeated measurements of several serum samples was <5.0%. The detection limit was 10 pg/mL. To evaluate bone formation, measurements of P1NP were performed on the Elecsys 2010 with a 2-site assay using monoclonal antibodies raised against intact human P1NP purified from human amniotic fluid. This assay detects both intact mono- and trimeric forms (total P1NP), as previously described [30]. The inter-assay CV was <3.0% and the lower detection limit <5 ng/mL.

### Statistical analysis

Subjects who reported either themselves or their parents or grandparents as being born outside Europe and North America, were excluded from the analysis to eliminate subjects with potential non-European ancestry. Subjects taking either anti-osteoporotic drugs or corticosteroids were also excluded. The bone measures; total hip and lumbar spine BMD_a_, calcaneal QUS, bone turnover markers and radial pQCT parameters were standardised: z = (x−μ)/)/σ where x = raw value of bone measure, μ = mean of bone measure and σ = standard deviation of bone measure. Linear regression was used to test for association between SNPs and these standardised bone measures under an additive genetic model adjusting for centre, height and weight. Results are reported as a percentage change (β) in a standard deviation (SD) with 95% confidence intervals (95%CI) for each copy of the minor allele. Significant associations were tested for heterogeneity between centres (using SNP*centre interactions) due to significant differences in bone measures and allele frequencies between centres. Associations with a p-value of <0.05 were considered statistically significant. Where multiple SNPs were significantly associated with a bone measure, conditional analysis was used to identify the SNPs independently associated (p<0.05) with the outcome.

The combined effect of multiple SNPs associated with the same bone measure was assessed by calculating a score for each subject that represented the total number of low BMD risk alleles carried. Linear regression adjusting for centre, height and weight was used to determine the association between the number of risk alleles (assuming linearity) and the bone measure. Results are reported as a percentage change (β) in a SD (95%CI) of the bone measure per one allele increase in the number of risk alleles.

SNPs were also tested for interaction under an additive model with E_2_ and free E_2_ as a continuous variable on measures of bone with which they were previously associated, adjusting for centre, height and weight. The effects of E_2_ and free E_2_ on the measure of bone by genotype (assuming linearity) and the interaction p-values are reported. All analysis was conducted in Stata version 9.2.

### Power

With 80% power and 5% type I error under an additive genetic model, the study was powered to detect effect sizes of greater than 0.2 SD for a minor allele frequency (MAF) = 0.05 and 0.1 SD for MAF = 0.5 for QUS BMD, bone turnover markers and E_2_. For DXA and pQCT measures, the study was powered to detect effect sizes greater than 0.36 SD for MAF = 0.05 and 0.16 SD for MAF = 0.5. Power was calculated using Quanto version 1.2.3 [31].

## Results

### Genotyping

**Figure 1:**
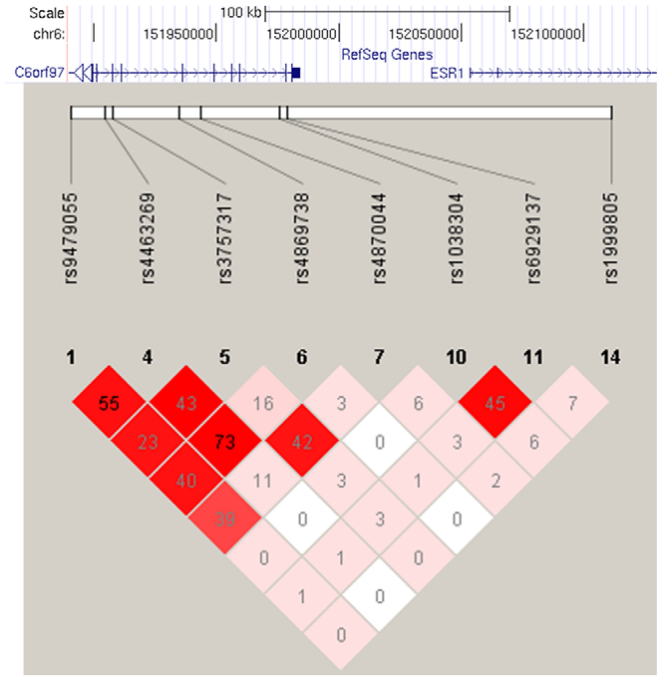
Linkage disequilibrium between genotyped *ESR1* (6q25) SNPs. Pair-wise values of D′ (coloured; white = 0, red = 1) and r^2^ (numbered) between SNPs genotyped in *ESR1* (6q25) are shown.

Eight SNPs from the study of Styrkarsdottir et al [14] were selected for genotyping in 2652 men who consented to genetic analysis and for whom ethical approval was obtained, of which 7 SNPs were successfully genotyped in 2584 (97%) subjects. Genotype data for rs3757317 was used as a proxy (r^2^ = 1, HapMap CEPH) for rs3734803. All SNPs had a genotyping success rate of >97% and HWE p>0.05. The LD between the SNPs in the EMAS population is shown in Figure 1.

### Subjects

In total, 2584 subjects were successfully genotyped. Subjects with non-European ancestry (n = 16) or using anti-osteoporotic drugs (n = 22) or corticosteroids (n = 78) were subsequently excluded resulting in 2468 subjects with a mean (SD) age of 59.9 (11.1) years from 7 centres with data on QUS, bone turnover markers and E_2_. A subset of these subjects (n = 628) also had data on DXA and pQCT measurements. The subject characteristics are shown in Table 1.

**Table 1:** Subject Characteristics.

Variable	Mean (SD)
Age at interview (years)	59.9 (11.1)
Total oestradiol (pmol/L)	74.0 (25.1)
Free oestradiol (pmol/L)	1.25 (0.43)
QUS	
Broadband attentuation (dB/MHz)	80.2 (19.1)
Speed of sound (m/s)	1551 (34.5)
Calcaneal eBMD (g/cm^2^)	0.54 (0.14)
Bone turnover	
β-cTX (ng/ml)	43.1 (21.5)
P1NP (ng/ml)	359.9 (181.9)
DXA	
Lumbar spine BMD_a_ (g/cm^2^)	1.06 (0.18)
Total hip BMD_a_ (g/cm^2^)	1.01 (0.14)
pQCT	
Cortical density (mg/cm^3^)	1155.9 (38.2)
Cortical BMC (mg/mm)	122.3 (17.2)
Total area (mm^2^)	149.6 (21.7)
Cortical thickness (mm)	3.24 (0.43)
Medullary area (mm^2^)	43.2 (17.6)
Cross-sectional muscle area (mm^2^)	3658.1 (632.9)
Stress strain index (mm^3^)	337.34 (64.84)
Total density (mg/cm^3^)	399.4 (72.3)
Total area (mm^2^)	375.8 (67.18)
Trabecular density (mg/cm^3^)	203.51 (43.2)

### The association between the *ESR1* (6q25) locus and bone health

Of the 8 SNPs tested, 5 were significantly associated with total hip BMD_a_ (Table 2). All five SNPs (rs3757317, rs4870044, rs1038304, rs6929137 and rs1999805) showed a similar size of effect; 0.12–0.15 SD lower total hip BMD_a_ for each copy of the minor allele. Two of these SNPs (rs1038304 and rs1999805) were also associated with lower BMD_a_ at the lumbar spine with the other three SNPs showing a trend towards an association with lower BMD_a_ at the lumbar spine that did not reach statistical significance. Three of these SNPs (rs4870044, rs1038304 and rs1999805) were also associated with lower ultrasound parameters at the calcaneus. The results were broadly similar for all QUS parameters (BUA, SOS and eBMD, which are highly correlated (Table S2), so the results presented here are for eBMD. The largest effect observed was for rs1999805 which showed a 0.11 SD (95%CI 0.05, 0.16) lower eBMD for each copy of the minor allele.

**Table 2:** Association between the *ESR1* (6q25) locus and QUS eBMD and DXA BMD_a_.

SNP	Chr position	Base change	MAF	Total hip BMD_a_		Lumbar spine BMD_a_		Calcaneal eBMD	
				β (95%CI)	p-value	β (95%CI	p-value	β (95%CI	p-value
rs9479055	151889660	A→C	0.41	−0.03 (−0.13, 0.07)	0.565	−0.01 (−0.12, 0.10)	0.848	−0.05 (−0.10, 0.01)	0.080
rs4463269	151903921	C→T	0.30	−0.00 (−0.11, 0.11)	0.957	−0.01 (−0.13, 0.11)	0.829	−0.01 (−0.07, 0.05)	0.637
rs3757317	151907224	C→T	0.15	−0.14 (−0.27, −0.01)	0.036	−0.09 (−0.23, 0.05)	0.221	−0.06 (−0.13, 0.02)	0.140
rs4869738	151933844	G→T	0.24	0.01 (−0.11, 0.12)	0.935	−0.02 (−0.15, 0.10)	0.714	−0.03 (−0.09, 0.04)	0.405
rs4870044	151943102	C→T	0.28	−0.12 (−0.23, −0.02)	0.021	−0.05 (−0.17, 0.06)	0.371	−0.10 (−0.15, −0.04)	0.002
rs1038304	151974868	A→G	0.49	−0.15 (−0.25, −0.05)	0.003	−0.11 (−0.22, −0.003)	0.045	−0.08 (−0.13, −0.02)	0.004
rs6929137	151978370	G→A	0.31	−0.13 (−0.24, −0.03)	0.013	−0.08 (−0.20, 0.03)	0.152	−0.05 (−0.11, 0.01)	0.131
rs1999805	152110057	T→C	0.41	−0.14 (−0.24, −0.05)	0.004	−0.12 (−0.23, −0.02)	0.026	−0.11 (−0.16, −0.05)	0.0001

β, effect estimates are shown as standardized values (standard deviations above or below the population average) for each copy of the minor allele adjusted for height and weight.

The association between SNPs in the *ESR1* (6q25) locus and bone health at the radius measured by pQCT is shown in Table 3. At the midshaft radius, the largest effect observed was for rs4870044 (one of the SNPs associated with lower total hip BMD_a_ and calcaneal eBMD) which showed a 0.19 SD (95%CI 0.07, 0.31) lower cortical density for each copy of the minor allele. Another SNP, rs9479055, (also associated with lower calcaneal eBMD) was associated with lower cortical density [0.13 SD (95%CI 0.01, 0.24)]. At the distal radius, two SNPs were significantly associated with trabecular density. Rs1999805 and rs1030304 were associated with a 0.18 SD (95%CI 0.06, 0.29) and a 0.14 SD (95%CI 0.03, 0.25) lower trabecular density for each copy of the minor allele, respectively. Both of these SNPs were also associated with lower total hip BMD_a_ and calcaneal eBMD. There was an association between a single SNP and geometric pQCT parameters at the radius. rs9479055 was associated with a decrease in cortical BMC [β = −2.18 (95%CI −3.99, −0.36) p = 0.019 per allele] and cortical thickness [β = −0.05 (95%CI −0.10, −0.01) p = 0.031 per allele]. This SNP was also associated with lower midshaft cortical density and there is moderate correlation between cortical density, BMC and thickness, r = 0.44–0.82 (Table S2) There was no association between any of the SNPs and markers of bone turnover, P1NP and β-cTX. There was no evidence of heterogeneity between centres for any of the significant associations.

**Table 3:** Association between the *ESR1* (6q25) locus and pQCT BMD.

SNP	Chr position	Base change	MAF	Midshaft radius cortical density		Distal radius trabecular density	
				β (95%CI)	p-value	β (95%CI)	p-value
rs9479055	151889660	A→C	0.41	−0.13 (−0.24, −0.01)	0.028	−0.07 (−0.19, 0.05)	0.266
rs4463269	151903921	C→T	0.30	−0.04 (−0.16, 0.09)	0.531	−0.01 (−0.14, 0.12)	0.890
rs3757317	151907224	C→T	0.15	−0.12 (−0.27, 0.03)	0.119	−0.05 (−0.21, 0.11)	0.562
rs4869738	151933844	G→T	0.24	−0.01 (−0.14, 0.13)	0.917	0.01 (−0.13, 0.15)	0.881
rs4870044	151943102	C→T	0.28	−0.19 (−0.31, −0.07)	0.002	−0.12 (−0.24, 0.01)	0.065
rs1038304	151974868	A→G	0.49	−0.03 (−0.14, 0.08)	0.604	−0.14 (−0.25, −0.03)	0.016
rs6929137	151978370	G→A	0.31	−0.01 (−0.13, 0.11)	0.842	−0.08 (−0.20, 0.04)	0.206
rs1999805	152110057	T→C	0.41	−0.08 (−0.19, 0.03)	0.148	−0.18 (−0.29, −0.06)	0.003

β, effect estimates are shown as standardized values (standard deviations above or below the population average) for each copy of the minor allele adjusted for height and weight.

In the conditional analysis the three SNPs associated with eBMD (rs4870044, rs1038304 and rs1999805), were shown to be independent of one another. Two SNPs, rs1038304 and rs1999805, were independently associated with total hip BMD_a_. Neither rs1999805 or rs1038304, were significantly independently associated with LS BMD_a_, however, the effect estimates remained similar (0.09 to 0.10 SD decrease per allele) to the individual SNP effects when combined in the same model. rs4870044 was significantly independently associated with cortical density and rs1999805 was significantly independently associated with trabecular density.

**Figure 2:**
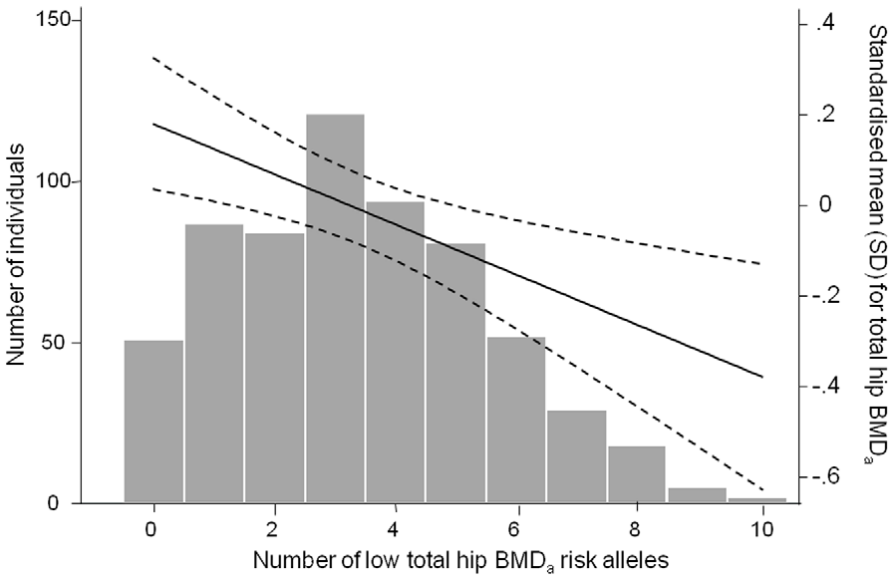
The association between the number of risk alleles and total hip BMD_a_. Histogram and line plot showing the combined effect of alleles associated with lower total hip BMD_a_. The line represents the standardised mean (SD) for total hip BMD_a_ by the number of low total hip BMD_a_ risk alleles. The dashed lines are the upper and lower bounds of the 95% confidence intervals of the mean.

To examine the potential combined effect of SNPs in *ESR1* (6q25) on bone health, a score was calculated (0–10) for each subject that represented the total number of risk alleles carried for the five SNPs associated with lower total hip BMD_a_ (rs1999805, rs1038304, rs4870044, rs6929137 & rs3757317). Increasing number of risk alleles was associated with a lower total hip BMD_a_ [−0.06 SD (95%CI −0.09, −0.03) p = 0.0002 per allele carried]. This is shown in figure 2 along with the distribution of risk allele carriage. Similar results were observed between increasing number of risk alleles and LS BMD_a_ [−0.09 SD (95%CI −0.16, −0.02) p = 0.01], calcaneal eBMD [−0.06 SD (95%CI −0.09, −0.04) p = 6.05×10^−6^], trabecular [−0.13 (95%CI −0.20, −0.05) p = 0.001] and cortical density [−0.09 SD (95%CI −0.16, −0.03) p = 0.005].

### The association between total / free E_2_ and bone density by *ESR1* (6q25) genotypes

As expected, increasing levels of both total and free E_2_ (pmol/l) were associated with higher BMD_a_ (expressed as SDs) at the hip (Total E_2_: β = 0.003 SDs per pmol/l; 95%CI = 0.0002, 0.006, Free E_2_: β = 0.32; 95%CI = 0.14, 0.50) and spine (Total E_2_: β = 0.005 SDs per pmol/l; 95%CI = 0.002, 0.008, Free E_2_: β = 0.36; 95%CI = 0.18, 0.53). Free E_2_ was also associated with higher QUS calcaneal eBMD (β = 0.33 ; 95%CI = 0.24, 0.42) and trabecular density (β = 0.23 ; 95%CI = 0.04, 0.42) at the radius as previously reported [9], [32] but did not reach a significant association with cortical density (β = 0.18 ; 95%CI = −0.01, 0.37) at the radius. None of the 8 SNPs were associated with either total or free E_2_ levels, however, when the positive association between total E_2_ and total hip BMD_a_ was stratified by the number of risk alleles of rs1038304, the strongest increase was observed in those with no risk alleles (β = 0.010; 95%CI = 0.003, 0.014), a smaller increase was observed in those with 1 risk allele (β = 0.002; 95%CI = −0.0005, 0.005) and there was no association in those with two risk alleles (β = −0.004; 95%CI = −0.008, 0.001). This interaction was statistically significant (p = 0.006). A similar interaction was observed between rs1038304 and E2 on LS BMD_a._(p = 0.025).

An interaction effect was also observed when the association between free E_2_ and total hip BMD_a_ was stratified by rs1038304 (p = 0.007) (Table 4). Interaction effects werealso observed when the associations between free E_2_ and LS BMD_a_ and radius trabecular density were stratified by rs1038304 alleles, Table 4.

**Table 4:** Association between free E_2_ and bone density by *ESR1* (*6q25*) genotypes.

Outcome	SNP	N copies of allele			p-value
		0	1	2	
		β (95%CI)	β (95%CI)	β (95%CI)	
Total hip BMD_a_	rs1038304	0.50 (0.19, 0.82)	0.17 (0.002, 0.34)	−0.17 (−0.44, 0.11)	0.007
Lumbar spine BMD_a_	rs1038304	0.58 (0.23, 0.93)	0.23 (0.05, 0.41)	−0.12 (−0.42, 0.18)	0.011
Trabecular density	rs1038304	0.54 (0.17, 0.91)	0.25 (0.05, 0.45)	−0.04 (−0.37, 0.28)	0.046

β, effect estimates are shown as standardized values (standard deviations) per pmol/L change in free E_2_; p-value is for the interaction.

## Discussion

Here we report, in a population sample of European Caucasian men, significant association between SNPs in the *ESR1* (6q25) locus and bone phenotypes at different anatomical sites using a variety of densitometric techniques. We confirm the findings of a previous GWAS, showing a significant association between this locus and total hip and lumbar spine BMD_a_ with very similar magnitude of effect (0.1 SD per copy of minor allele, Table S1). In addition, we observed association between several of the SNPs and calcaneal ultrasound eBMD of similar magnitude to that of BMD_a_. We also observed associations with cortical and trabecular density at the radius. Only 2 of the 8 SNPs were associated with lumbar spine BMD_a_ but others showed an effect in the same direction but just failed to attain statistical significance which may be due to the relatively small sample size compared to the GWAS or possibly technical artefact as BMD_a_ of the spine can be artificially raised in an elderly population by concomitant degenerative disc disease and osteoarthritis, potentially disguising any real influence these SNPs may have on bone density.

To our knowledge this is the first study to examine the influence of the *ESR1* (6q25) locus on calcaneal QUS parameters. We found associations between the SNPs and higher QUS parameters, of similar magnitude to that of total hip BMD_a_. We previously investigated four other SNPs in *ESR1* (rs488133, rs2077647, rs1801132 and rs726282) for an association with calcaneal QUS parameters. These showed only very modest evidence of association in heterozygous individuals only [33]. However, there is no LD between these four SNPs and the SNPs investigated in this analysis. The associations reported here are much stronger and taken with the previously reported associations with BMD_a_ at the genome-wide significance level, suggests that the 6q25 locus is an important locus in determining bone health.

In our study, we observed that rs9479055 and rs4870044 were associated with higher cortical density, and rs1038304 and rs1999805 were associated with higher trabecular density at the radius. Few studies have examined the influence of this genetic locus on volumetric bone density or bone geometry. A study of 3113 boys and girls from the Avon Longitudinal Study of Parents and Children (ALSPAC) found that rs1038304 was associated with cortical BMD at the tibia [34]. This study did not examine any of the other *ESR1* (6q25) SNPs or the trabecular compartment of the tibia. ALSPAC found no association with any of the geometric parameters [34], in keeping with our findings and tending to suggest that the effect of 6q25 on BMD is not via an influence on skeletal size.

While our data replicate previous associations found between SNPs in the 6q25 locus and BMD_a_ at the hip and extend these data to include associations with calcaneal ultrasound parameters and radial volumetric BMD, we observed no associations with the markers of bone turnover P1NP and β-cTX. In older men, increased bone turnover markers have been shown to be associated with lower BMD_a_ and, more recently, poor bone microarchitecture [35], [36]. In line with these cross-sectional findings, prospective data have confirmed that higher levels of bone remodeling may be associated with increased rates of bone loss, although evidence for an increased risk of fracture is lacking [37]–[39]. Our results suggest that the effect on bone health of the individual SNPs studied in this analysis was independent of an effect on bone remodeling. To our knowledge, no other studies have examined this relationship. Previous studies have investigated other regions of *ESR1* and bone turnover but have produced conflicting results [12].The reasons for this remain to be clarified.

Examining our results overall, three SNPs in particular were consistently associated with bone health. Rs1038304 and rs1999805 were associated with higher hip BMD_a_, calcaneal QUS and trabecular density at the distal radius. Rs4870044 was associated with higher hip BMD_a_, calcaneal QUS and cortical density at the mid-shaft radius. These three SNPs span a 220 kb region, with rs1038304 and rs4870044 being located in intronic regions of *C6orf97* (a gene of unknown function upstream of *ESR1*) and rs1999805 is located within intron 2 of a splice variant of *ESR1*. We showed that these three SNPs were independently associated with eBMD, the BMD measure for which the largest sample size was available. For the other BMD measures these SNPs were not always significantly independent, however, the effect estimates remained similar suggesting that the lack of ability to identify independent associations was due to the reduced sample size for the DXA and pQCT measures of BMD. In addition, LD between these SNPs is low and Stykarsdottir et al. [14] also observed that independent associations with bone density exist at this locus. Recent studies have also identified significant differences between European and East Asian populations at this locus for DXA BMD with large differences in allele frequencies observed for many SNPs [40], [41]. Furthermore, these SNPs are not in LD with the three most widely studied polymorphisms in *ESR1*, namely rs2234693, rs9340799 and the (TA)_n_ variable number tandem repeat [12], indicating that the pattern of association across *ESR1* is complex. The fact that we observed associations across a number of anatomical sites irrespective of the densitometric technique used provides further evidence that 6q25 is an important genetic determinant of bone health.

Interestingly, we observed that the expected positive relationship between serum levels of total / free E_2_ and hip BMD_a_ and our previously reported association between free E_2_ and distal radius trabecular density [32] were moderated by rs1038304. A weak interaction was also seen with rs6929137. This suggests that these SNPs may influence the ability of *ESR1* to mediate the action of oestradiol. These interactions, however, require replication in independent populations to determine if this is a true effect.

EMAS is a large, multi-centre population based study using standardised methods to assess both genetic variation and related phenotypes. However, there are a number of limitations that need to be considered when interpreting the results. The overall response rate for participation was 45%, and it is possible that those invited but declined to participate may have differed from those who agreed to participate. Caution is required therefore in interpreting absolute values; both the frequency of individual SNPs and the phenotype data. However, given that the main analysis was based on an internal comparison of the participants, it is unlikely that any selection factors would have influenced the main findings. In any multi-centre study, there is the possibility of population stratification. In our study there was no evidence of any between centre heterogeneity and the likelihood of population stratification was minimised by excluding subjects of non-European ancestry. False-negative results may have occurred owing to power constraints, particularly in relation to the findings for DXA and pQCT. False positive associations might have occurred due to multiple testing as only a single association (rs1999805 and calcaneal eBMD) reached a bonferroni corrected p-value threshold of 0.00037, however, this threshold does not account for the correlation between bone measures (Table S2). However, the number of SNPs examined was relatively small and these SNPs have been shown to be associated with BMD_a_ at the genome-wide significance level in previous studies.

We observed the effect of each individual SNP on bone health to be modest. This is in keeping with the majority of studies examining the contribution of genetic factors to bone health. Individual SNPs may not be useful in predicting individuals at risk of developing osteoporosis. However, it remains possible these risk markers may contribute to a genetic risk profile that in combination with environmental predictors might help identify at-risk individuals. Analysing the combined effects of the associated SNPs in the locus showed that compared to subjects with no risk alleles, those with all the risk alleles had a considerably lower BMD. This suggests that the additive effect of SNPs in this locus may have a large impact on BMD in a small number of individuals. However, limiting the analysis to those SNPs associated with BMD may have introduced bias. We also analysed the combined effect of all SNPs genotyped on each of the BMD measures (data not shown). Except for LS BMD_a_, the effect was significant for all BMD measures. The effect sizes, however, were much smaller e.g. for total hip BMD_a_ the combined effect of all SNPs tested (per allele) was half of the combined effect of the SNPs associated with total hip BMD_a_. Further work is required to identify the full complement of genetic predictors of bone health and how they interact with each other and non-genetic risk factors.

In conclusion, our data replicate previous associations found between SNPs in the *ESR1* (6q25) locus and BMD_a_ at the hip and extend these data to include associations with calcaneal ultrasound parameters and radial volumetric BMD. If these findings are confirmed in other independent populations, fine mapping and functional studies may lead to identification of causal variants and their modes of action. The significance of these findings in relation to male osteoporosis also merits further study.

## Supporting Information

Table S1 **The combined effect (discovery and validation cohorts) for the SNPs tested in Styrkarsdottir et al., 2008.**
(DOCX)

Table S2 **Correlation coefficients between outcome variables.**
(DOCX)
