# Supplementary material for: The ESR1 (6q25) Locus Is Associated with Calcaneal Ultrasound Parameters and Radial Volumetric Bone Mineral Density in European Men
Source: PLoS One. 2011 Jul 7;6(7):e22037. doi: 10.1371/journal.pone.0022037 (PMC3131390; doi:10.1371/journal.pone.0022037)
Supplement: Table S1 — The combined effect (discovery and validation cohorts) for the SNPs tested in Styrkarsdottir et al., 2008. (DOCX) [file pone.0022037.s001.docx]

**Table S1. The combined effect (discovery and validation cohorts) for the SNPs tested in Styrkarsdottir et al., 2008.**

| **SNP** | **Chr position** | **Base change** | **MAF** | **Hip BMD_a_** | | **LS BMD_a_** | |
| --- | --- | --- | --- | --- | --- | --- | --- |
|  |  |  |  | ** β (95%CI)** | **p-value** | ** β (95%CI)** | **p-value** |
| rs9479055 | 151889660 | A→C | 0.35 | -0.08 (-0.11, -0.05) | 3.05 x 10^-8^ | -0.08 (-0.11, -0.05) | 6.22 x 10^-7^ |
| rs4463269 | 151903921 | C→T | 0.25 | -0.08 (-0.11, -0.05) | 6.76 x 10^-7^ | -0.07 (-0.10, -0.03) | 5.00 x 10^-5^ |
| rs3734803* | 151911338 | C→T | 0.16 | -0.09 (-0.13, -0.05) | 3.16 x 10^-6^ | -0.09 (-0.13, -0.06) | 1.68 x 10^-6^ |
| rs4869738 | 151933844 | G→T | 0.18 | -0.08 (-0.12, -0.05) | 4.74 x 10^-6^ | -0.08 (-0.12, -0.04) | 1.30 x 10^-5^ |
| rs4870044 | 151943102 | C→T | 0.28 | -0.08 (-0.11, -0.05) | 1.56 x 10^-7^ | -0.11 (-0.14, -0.08) | 1.62 x 10^-11^ |
| rs1038304 | 151974868 | A→G | 0.47 | -0.08 (-0.11, -0.06) | 5.26 x 10^-9^ | -0.10 (-0.13, -0.07) | 3.97 x 10^-11^ |
| rs6929137 | 151978370 | G→A | 0.30 | -0.08 (-0.11, -0.05) | 1.03 x 10^-7^ | -0.10 (-0.13, -0.07) | 2.45 x 10^-10^ |
| rs1999805 | 152110057 | T→C | 0.44 | -0.06 (-0.09, -0.03) | 1.20 x 10^-4^ | -0.09 (-0.12, -0.06) | 2.22 x 10^-8^ |

β, effect estimates are shown as standardized values (standard deviations above or below the population average) for each copy of the minor allele, BMD was adjusted for age and weight.

*rs3757317 (r^2^=1, HapMap CEPH) was genotyped in EMAS
