# Supplementary material for: The ESR1 (6q25) Locus Is Associated with Calcaneal Ultrasound Parameters and Radial Volumetric Bone Mineral Density in European Men
Source: PLoS One. 2011 Jul 7;6(7):e22037. doi: 10.1371/journal.pone.0022037 (PMC3131390; doi:10.1371/journal.pone.0022037)
Supplement: Table S2 — Correlation coefficients between outcome variables. (DOCX) [file pone.0022037.s002.docx]

**Table S2. Correlation coefficients between outcome variables**

|  | | **QUS** | | | **DXA** | | **pQCT 50%** | | | | | | | **pQCT 4%** | | | **Turnover** | |
| --- | --- | --- | --- | --- | --- | --- | --- | --- | --- | --- | --- | --- | --- | --- | --- | --- | --- | --- |
|  |  | **eBMD** | **BUA** | **SOS** | **LS BMD_a_** | **Total Hip BMD_a_** | **Cortical density** | **Cortical BMC** | **Total area** | **Cortical thickness** | **Medullary area** | **Cross-sectional muscle area** | **Stress strain index** | **Total density** | **Total area** | **Trabecular density** | **β-cTX** | **P1NP** |
| **QUS** | **eBMD** | 1.00 |  |  |  |  |  |  |  |  |  |  |  |  |  |  |  |  |
|  | **BUA** | 0.95 | 1.00 |  |  |  |  |  |  |  |  |  |  |  |  |  |  |  |
|  | **SOS** | 0.98 | 0.89 | 1.00 |  |  |  |  |  |  |  |  |  |  |  |  |  |  |
| **DXA** | **LS BMD^a^** | 0.46 | 0.45 | 0.45 | 1.00 |  |  |  |  |  |  |  |  |  |  |  |  |  |
|  | **Total Hip BMD^a^** | 0.51 | 0.49 | 0.51 | 0.68 | 1.00 |  |  |  |  |  |  |  |  |  |  |  |  |
| **pQCT 50%** | **Cortical density** | 0.22 | 0.18 | 0.24 | 0.19 | 0.27 | 1.00 |  |  |  |  |  |  |  |  |  |  |  |
|  | **Cortical BMC** | 0.27 | 0.25 | 0.27 | 0.42 | 0.43 | 0.44 | 1.00 |  |  |  |  |  |  |  |  |  |  |
|  | **Total area** | 0.04 | 0.05 | 0.03 | 0.22 | 0.14 | -0.36 | 0.45 | 1.00 |  |  |  |  |  |  |  |  |  |
|  | **Cortical thickness (mm)** | 0.27 | 0.25 | 0.27 | 0.34 | 0.40 | 0.58 | 0.82 | -0.07 | 1.00 |  |  |  |  |  |  |  |  |
|  | **Medullary area** | -0.14 | -0.13 | -0.15 | -0.05 | -0.14 | -0.62 | -0.2 | 0.77 | -0.68 | 1.00 |  |  |  |  |  |  |  |
|  | **Cross-sectional muscle area** | 0.09 | 0.09 | 0.08 | 0.17 | 0.31 | -0.04 | 0.38 | 0.35 | 0.24 | 0.09 | 1.00 |  |  |  |  |  |  |
|  | **Stress strain index** | 0.14 | 0.14 | 0.13 | 0.30 | 0.26 | -0.02 | 0.73 | 0.79 | 0.32 | 0.35 | 0.44 | 1.00 |  |  |  |  |  |
| **pQCT 4%** | **Total density** | 0.34 | 0.36 | 0.33 | 0.31 | 0.40 | 0.23 | 0.20 | -0.08 | 0.30 | -0.24 | 0.04 | -0.05 | 1.00 |  |  |  |  |
|  | **Total area** | -0.04 | -0.06 | -0.02 | 0.09 | 0.05 | 0.01 | 0.37 | 0.44 | 0.12 | -0.23 | 0.32 | 0.50 | -0.61 | 1.00 |  |  |  |
|  | **Trabecular density** | 0.47 | 0.43 | 0.49 | 0.36 | 0.46 | 0.27 | 0.27 | -0.04 | 0.32 | 0.14 | 0.08 | 0.04 | 0.60 | -0.15 | 1.00 |  |  |
| **Turnover** | **β-cTX** | -0.09 | -0.04 | -0.10 | -0.12 | -0.13 | -0.18 | -0.15 | 0.05 | -0.18 | 0.15 | -0.03 | 3E-04 | -0.09 | -0.03 | -0.14 | 1.00 |  |
|  | **P1NP** | -0.04 | -0.02 | -0.06 | -0.02 | -0.06 | -0.14 | -0.08 | 0.09 | -0.14 | 0.09 | -0.03 | 0.07 | -0.09 | 0.04 | -0.08 | 0.61 | 1.00 |
